# Linkages of agroecosystems producing farmed seafood on food security, nutritional status and adolescent health in Bangladesh

**DOI:** 10.1111/mcn.13017

**Published:** 2020-12-21

**Authors:** Baukje de Roos, Nanna Roos, Gulshan Ara, Tahmeed Ahmed, Abdullah‐Al Mamun, Alan A. Sneddon, Francis Murray, Eleanor Grieve, David C. Little

**Affiliations:** ^1^ The Rowett Institute University of Aberdeen Aberdeen UK; ^2^ Department of Nutrition, Exercise and Sports University of Copenhagen Copenhagen Denmark; ^3^ Nutrition and Clinical Services Division International Centre for Diarrhoeal Disease Research Dhaka Bangladesh; ^4^ Department of Fisheries and Marine Science Noakhali Science and Technology University Noakhali Bangladesh; ^5^ Institute of Aquaculture University of Stirling Stirling UK; ^6^ Institute of Health and Wellbeing University of Glasgow Glasglow UK

**Keywords:** adolescent nutrition, aquaculture, behaviour, developing countries, farmed seafood, food and nutrient intake, food security, food systems, malnutrition, nutritional status

## Abstract

This narrative review aims to provide an interdisciplinary perspective on actors that link global aquatic food production systems with fish consumption and nutritional status, with a special focus on adolescent girls in Bangladesh. The writing of this narrative perspective was undertaken within the framework of the Aquatic Food for Health and Nutrition (AQN) project that aimed to develop a metric for assessing the impacts on nutrition and health of agroecosystems producing farmed seafood. Previous studies evaluating links between agricultural ecosystems, aquaculture, food security and human health have systemically ignored the importance of diet and nutrition. Such studies have also ignored the importance of local communities, cultural norms and household composition and behaviours to identify vulnerable groups such as adolescent girls. This narrative review presents our current understanding of the relationships between aquaculture, fish production and consumption patterns, food security, optimal nutrition and health. It also highlights the importance of research into aquaculture food systems, linking aquatic food production systems with nutritional status, health and socioeconomic prosperity, which would help to develop more integrated and relevant food policies.

**Key messages**

- Aquaculture plays a key role in food security and protein intake, especially in low‐ and middle‐income countries.
- Previous studies evaluating links between agricultural ecosystems, aquaculture, food security and human health have systemically ignored the importance of diet and nutrition, local communities, cultural norms and household composition and behaviours.
- We are in need of more integrated and relevant aquaculture food policies that link aquatic food production systems with nutritional status, health and socioeconomic prosperity.
- Polices aiming to improve nutrition and health outcomes in adolescent girls are of particular importance, as an improvement in nutritional status will affect not only their own current and future health but also that of the next generation of children.

## 1 INTRODUCTION

Diet plays a significant role in the maintenance of health worldwide, and improvement of diets could potentially prevent one in every five deaths globally (GBD 2017 Diet Collaborators, 2019). Malnutrition affects one in three people globally and presents one of the main health challenges we are currently facing (International Food Policy Research Institute Global Nutrition Report, 2016). Increasingly, cardiometabolic risk factors such as high blood pressure, blood glucose, serum cholesterol and overweight add to the mortality burden in low‐ and middle‐income countries, and prevalence of maternal overweight now exceeds that of underweight in all those regions (Black et al., 2013). The double burden of undernutrition and increasing problems with overweight, obesity and chronic diseases contribute to maternal and child malnutrition and mortality in low‐ and middle‐income countries (Black et al., 2013; Global Burden of Metabolic Risk Factors for Chronic Diseases Collaboration, 2014).

Although many observational and intervention studies have significantly enhanced our understanding of associations between nutrients, diets, nutrition and health outcomes, it has been argued that sustainable strategies to tackle malnutrition and food inequalities should consider the entire food system. Thus, the link between diet and health should be considered in parallel to food production, processing and marketing, purchasing and eating behaviours as well as environmental impact of diets. Furthermore, we should also consider socioeconomic, societal and political contexts (Dangour, Mace, & Shankarbd, 2017). Indeed, scholars have been aware for some time that increasing incomes and urbanisation, as well as population growth, are important factors that affect lifestyles, food consumption patterns and agri‐food systems. Increased disposable incomes have already caused a shift towards purchase and consumption not only of higher value items such as fish, meat, dairy products and fruits (Gerbens‐Leenes, Nonhebel, & Krol, 2010; Mottaleb, Rahut, Kruseman, & Erenstein, 2017) but also towards highly processed convenience foods favoured particularly by the young (Pries, Filteau, & Ferguson, 2019) in Bangladesh and in a range of other countries.

Access to fish plays a key role in food security and population health, and global fish supply is increasingly dependent on aquaculture production systems (Food and Agriculture Organization of the United Nations [FAO], 2018). Although global capture production has been relatively static since the 1980s, the supply of fish for human consumption from aquaculture has grown significantly from 7% in 1974 to providing 53% of fish for direct human consumption in 2016 (FAO, 2018). In low‐ and middle‐income countries, the contribution of aquaculture to national GDP varies from almost 0% in countries where the sector is emerging, like India, Kenya and Zambia, up to 5% or more in countries where the sector is very dynamic, such as Bangladesh and Vietnam (Aquaculture for Food Security, Poverty Alleviation and Nutrition, 2015). However, studies evaluating the role of aquaculture on population health in Asia, Africa and South America have systemically ignored the importance of diet and nutrition (Burns, Wade, Stephen, & Toews, 2014), as well as other drivers of food consumption such as the importance of local communities, cultural norms and household composition (de Roos et al., 2019). The contribution of fish and seafood to the intake of energy from animal‐based food is significantly higher in Bangladesh than in four South East Asian countries known for significant consumption (China, India, Philippines and Vietnam). Moreover, the contribution of fish and seafood, and particularly fresh water fish, to dietary energy, fat and protein intakes across these South East Asian countries is also among the highest in Bangladesh (de Roos et al., 2019). In this context, the contribution of fish and seafood to nutritional status in the Bangladeshi population is significant, and changes to the supply and accessibility of aquatic foods are therefore likely to impact on dietary quality.

This narrative review presents our current understanding of the role of aquaculture food systems, which includes smallholders, larger farmers, domestic and international supply, production and processing systems and government investments, on fish production and consumption patterns and on maintaining food security and overall population health. We will introduce arguments as to why a better understanding of access to aquatic foods, nutritional status and well‐being should consider intrahousehold distribution and consumption. This review will focus specifically on fish consumption in adolescent girls, a recognised vulnerable group in low‐ and middle‐income countries such as Bangladesh (World Health Organization, 2017).

## 2 METHODS

**FIGURE 1:**
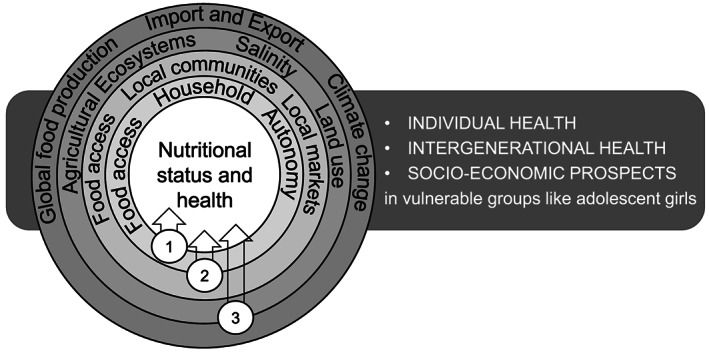
Conceptual framework outlining the global, local and household determinants of nutritional status and its link to individual health, intergenerational health and socioeconomic prospects. The Results and Discussion part of this review is based on the three major layers of the conceptual framework, that is, (1) linking fish consumption, nutritional status and health, (2) linking access to fish in local communities and in households to optimal nutrition and health, and (3) linking agricultural ecosystems to nutritional status

Here, we present a narrative review discussing evidence obtained from academic papers and reports, and from the most up‐to‐date government and nongovernment organisation (NGO) reports, published in interdisciplinary research fields including agroecosystems producing farmed seafood, food security and nutrition published in the past 20 years (2000 to February 2020). The review was conducted by an interdisciplinary group of authors who were involved in the Immana‐funded Aquatic Food for Health and Nutrition (AQN) project (December 2017 to December 2019) that aimed to develop an integrated metric for assessing the impacts on nutrition and health of agroecosystems producing farmed seafood in adolescent girls in Bangladesh, based on the best predictors, including social and geographical factors contained within specific farmed seafood‐producing agroecosystems, of nutritional status and the omega‐3 index. This review explores relationships between aquaculture, agricultural ecosystems, access to food in local communities and in households and optimal nutrition and health (Figure 1). The paper focusses on adolescent girls in Bangladesh, a large and vulnerable cohort within the Bangladeshi population with a high prevalence of poor nutritional status because of poor diets and early childbearing (World Bank Group, 2019).

## 3 RESULTS AND DISCUSSION

The link between fish consumption and health outcomes is becoming increasingly clear (Abdelhamid et al., 2018; Food and Agriculture Organization of the United Nations & World Health Organization [FAO/WHO], 2010). Furthermore, the role of fisheries and aquaculture supply and value chains in the provision of employment, and in securing livelihoods of poor households, is well established (Allison, 2011; Béne et al., 2016). However, the actual contribution of the national aquaculture and fishery industries to food security, fish consumption and the nutritional status of its inhabitants is largely unknown. We will discuss our knowledge of global, local and household determinants of nutritional status and its link to individual health, intergenerational health and socioeconomic prospects based on the conceptual framework presented in Figure 1.

### 3.1 Linking fish consumption, nutritional status and health

The beneficial effects of seafood consumption on health, which includes a lowering in the risk of mortality from coronary heart disease in Western populations, have traditionally been attributed to its content of long chain n‐3 polyunsaturated fatty acids (LC n‐3 PUFA; FAO/WHO, 2010). However, a recent systematic assessment of the effects of LC n‐3 PUFA, mostly provided as fixed‐dose supplements, on cardiovascular health outcomes indicated that increasing consumption had little or no effect on mortality or cardiovascular health. It was hypothesised that previous suggestions of benefits from LC n‐3 PUFA supplements appear to arise from trials with a higher risk of bias (Abdelhamid et al., 2018). Nevertheless, the review reiterated the health benefits of fish consumption, and indeed, the health effects of fish consumption would be greater than the sum of its individual constituents such as LC n‐3 PUFA (de Roos, Sneddon, Sprague, Horgan, & Brouwer, 2017). Two meta‐analyses of fish intervention studies have confirmed that compared with very low fish intake (i.e., less than one serving per month), low fish intake (one serving per week) reduces risk for coronary heart disease and stroke by 16% and 14%, respectively, and moderate fish intake (two to four servings per week) reduces risk for coronary heart disease and stroke by 21% and 9%, respectively (Xun et al., 2012; Zheng et al., 2012).

The importance of fish consumption for nutritional status and health outcomes appears to be of higher significance in low‐ and middle‐income countries. In 2010, of the 30 countries where fish contribute more than one third of the total animal protein intake, 22 were in low‐ and middle‐income countries (Kawarazuka & Béné, 2010). In these countries, fish are an important and often exclusive source of micronutrients, vitamins and LC n‐3 PUFA (Golden et al., 2016). Several studies indicate that fish consumption plays an important role in child development and growth. Indeed, the role of dietary LC n‐3 PUFA in the development of brain and retina of infants and children is well documented (Lauritzen, Hansen, Jørgensen, & Michaelsen, 2001), and maternal seafood consumption has been positively associated with weight and head circumference of babies at birth in the Norwegian Mother and Child Cohort Study (Brantsæter et al., 2012). Increasing access to high‐quality proteins from fish, and also from meat, pork and milk products, in addition to generally higher standards of living, better healthcare, lower children's mortality, lower fertility rates, higher levels of urbanisation and higher social equality, has been associated with the height of young men across 45 countries as a health outcome (Grasgruber, Cacek, Kalina, & Sebera, 2014).

Fish is also a valuable contributor to the reference nutrient intakes for a range of micronutrients, and therefore, fish consumption may contribute to alleviating highly prevalent micronutrient deficiencies (Bogard, Marks, Mamun, & Thilsted, 2017b; Roos, Wahab, Chamnan, & Thilsted, 2007). In Bangladesh, for example, the mean intake per adult male equivalent per day from fish was 1.6 mg for iron, 1.0 mg for zinc, 279 mg for calcium, 47.8‐μg retinol activity equivalent (RAE) for vitamin A and 1.3 μg for vitamin B_12_ in 2010 (Bogard et al., 2017a), representing a significant contribution towards the RNI (FAO/WHO, 2004): up to 15% for iron, 14% for zinc, 28% for calcium, 8% for vitamin A and 54% for vitamin B_12_. In this respect, it is important to highlight that the promotion of the consumption of mola carplet (*Amblypharyngodon mola*), a small indigenous fish high in vitamin A, appeared a cost‐effective approach to increase vitamin A intake, reduce the prevalence of inadequate vitamin A intake and generally reduce the burden of micronutrient malnutrition in Bangladesh (Fiedler, Lividini, Drummond, & Thilsted, 2016). It was recently calculated that 1.39 billion people worldwide (equivalent to 19% of the global population) would be vulnerable to deficiencies in certain nutrients if fish stocks are insufficient to feed populations in the future (Golden et al., 2016). This global model was limited to marine fisheries, but with rising pressures on this sector, demand for fish is increasingly being met from aquaculture. It is interesting to see that the current Bangladeshi policy setting out strategies to improve the overall health, nutritional status, growth and development by preventing and alleviating micronutrient deficiencies actually addresses the impact nutrition‐sensitive agriculture and food systems for promoting food security (National Strategy on the Prevention and Control of Micronutrient Deficiencies Bangladesh, [2015‐2024], 2015).

While all fish are important sources of nutritiously high‐quality protein and fat, access to small fish species specifically are important as these contribute important minerals and vitamins to the diets. Small fish are consumed mostly as a whole, with bones, guts and intestines, and edible portions therefore include bones rich in bioavailable calcium and phosphate and tissues rich in iron and potentially vitamin A (Bogard et al., 2017a; Roos et al., 2007; Roos, Islam, & Thilsted, 2003). In that respect, the nutritional equivalency of larger stocked fish in diets, lacking in key micronutrients, compared with that of small indigenous species, which they have to some extent replaced, has been challenged (Bogard et al., 2017a; Bogard et al., 2017b).

### 3.2 Linking access to fish in local communities and in households to optimal nutrition and health

It is unclear exactly how aquaculture production systems contribute to the population and individual health and well‐being and how this relationship can be affected by food availability, dietary intakes and nutritional status on the local, household and individual level. We currently lack food system approaches that are necessary to create a better understanding of impacts of access to aquatic foods on health and nutrition, as well as product attributes, that underpin purchasing behaviours of especially poorer consumers. Research on metrics for the linkages between aquaculture and terrestrial agroecosystems and nutritional and health outcomes in Bangladesh have already highlighted the need for more research on in‐country‐specific settings, including dietary diversity and the role of women in food production and distribution (de Roos et al., 2019). Evidence for improved access to fish and other sources of micronutrients on nutritional and health outcomes has been limited and contradictory. One recent randomised controlled trial in Cambodia did find positive impacts of enhanced homestead food production including a fish pond to improve iron, vitamin A and riboflavin among women, but not among children, for example (Michaux et al., 2019).

Population growth, rising incomes, urbanisation and a strong expansion of global production and distribution of fish and fish products have led to a significant increase in the total supply of fish for food consumption in the past five decades (FAO, 2018). The demand for fish is projected to grow further in the next two decades and will be increasingly dependent on aquaculture (World Bank, 2013). Fish and products derived from international and local systems play an important role in providing a variety of important nutrients such as protein, LC n‐3 PUFA, vitamins and minerals (de Roos et al., 2017; de Roos et al., 2019). The way fish consumption may contribute to nutritional security for households engaged in small‐scale fisheries in low‐ and middle‐income countries is hypothesised to include the provision of important nutrients such as vitamin A, calcium, iron and zinc from consuming some of the fish they capture or farm, increase purchasing power through the sale of fish and enhance economic status and budget control for women through their involvement in aquaculture and fisheries‐related activities, such as fish processing and trading. However, it has already been noted that evidence for such pathways is often anecdotal and more research is required in this area (Kawarazuka & Béné, 2010).

Societal and cultural factors are important in household and individual food security and nutritional deficiency issues. For example, there are substantial inequities in intrahousehold distribution of calories and nutrients in Bangladesh. Although in lower economic well‐being households, male heads consume disproportionately large shares of calories and nutrients, women's disempowerment is associated with lower calorie and nutrient intake, which may have direct consequences for their nutritional status (D'Souza & Tandon, 2019). It has been argued that female autonomy plays an important role in food access and food choice, nutritional status and health in Ghana (Amugsi, Lartey, Kimani, & Mberu, 2016), and schooling and voice with husband was correlated with dietary diversity in a study in Bangladesh (Sinharoy et al., 2018). Improving female decision‐making autonomy could therefore have a positive impact on dietary intake in females and in their families. With Bangladesh already having significant rates of malnutrition, mainly resulting from inadequate dietary intake of animal foods, as well as fruit and vegetables (Black et al., 2013; Icddr,b, UNICEF, GAIN, IPHN, 2013), the group of adolescent girls may be particularly vulnerable to dietary deficiencies (National Strategy for Adolescent Health 2017–2030, 2016). Such deficiencies will not only impact on their own health but also on their children's health, as well as their countries' economic and social prospects (Patton et al., 2016). Indeed, adolescence is increasingly being recognised as a crucial life stage where individuals are especially vulnerable to nutrition‐related health threats such as infections, diarrhoeal diseases and iron‐deficiency anaemia (WHO, 2017).

The health of adolescents has improved far less than that of younger children over the past 50 years, and information on adolescent nutrition and health in Bangladesh is limited (World Bank Group, 2019). The overall dietary knowledge in this age group is low, with more than a third not being aware of the importance of taking extra nutrients during adolescence for growth spurt (Alam, Roy, Ahmed, & Ahmed, 2010). Data from the Food Security and Nutrition Surveillance Project in Bangladesh have highlighted a decrease in dietary diversity in adolescent girls between 2012 and 2014, with over 60% of these girls falling into the poor dietary diversity group. And whereas levels of stunting decreased from 17% to 11% in young adolescent girls, rates of overweight and obesity in older adolescent girls increased from 13% to 23% during this period, indicating substantial nutritional deficits relative to healthy norms in the adolescent period (World Bank Group, 2019). The shift in dietary intake in adolescent girls in low‐ and middle‐income countries from traditional to more Westernised diets will have a major impact on the double burden of malnutrition, as well as the rising prevalence of noncommunicable diseases. Although nutrient requirements for adolescent girls are high because of their development and growth, or to support the fetus in case of pregnancy, less than half of the girls in low‐ and middle‐income countries consume dairy products or meats/fish, whereas in those that do consume fruits and vegetables, they are not meeting their dietary guidelines for dairy, meat and fish and fruits and vegetables (Keats et al., 2018). An added problem is that there are very few guidelines that specifically target adolescent girls (Lassi et al., 2017), partly because of a lack of good‐quality, nationally representative data on which to base recommendations for adolescent nutrition in low‐ and middle‐income countries (Keats et al., 2018).

Strategies that place the adolescent years centre stage should be more prominent within future global public health policies and programming (GBD 2017 Diet Collaborators, 2019). The promotion of fish consumption as a main dietary source for protein, micronutrients and vitamins could play a key role in nutritional security in this vulnerable group. Fish consumption has been associated with improved academic and cognitive performance in adolescent boys in Sweden (Äberg et al., 2009; Kim et al., 2010), and with vocabulary and end‐term grades in adolescent boys and girls in the Netherlands (de Groot, Ouwehand, & Jolles, 2012) in observational studies. Furthermore, higher levels of eicosapentaenoic acid (EPA), a key LC n‐3 PUFA, in adipose tissue have been linked to fewer depressive symptoms in adolescent participants in Crete (Mamalakis et al., 2006). Therefore, any changes to the supply and accessibility of aquatic foods are likely to impact on the dietary quality of this population group, as well as on population malnutrition, cardiovascular and mental disease outcomes (de Roos et al., 2019).

### 3.3 Linking agricultural ecosystems to nutritional status

Aquaculture already contributes significantly to fish production and to gross domestic product in many South Asian countries, such as Bangladesh (Cai, Huang, & Leung, 2019; Little, Newton, & Beveridge, 2016). Bangladesh is considered as one of the most suitable regions for aquaculture and fisheries in the world, playing an important role in the economy and the diet of the population. This country has the world's largest area of flooded wetland suitable for aquaculture production systems and the third largest aquatic biodiversity in Asia behind only China and India (Ghose, 2014). Altered weather patterns and rising sea levels because of climate change have already led to increased sea/freshwater flooding and saline contamination of soils, especially in coastal regions in Bangladesh. Although inundation has had negligible effects on migration and agricultural production per se, it has actually increased diversification into aquaculture (Chen & Mueller, 2018). In Bangladesh, the growth in aquaculture has been coined a ‘quiet revolution’, largely based on commercially orientated family enterprises that focus on fish production and the fish value chain as a whole (Hernandez et al., 2018), thereby responding to the demand of a rapidly urbanising population (World Bank, 2018). Although globally, over 50% of global seafood production is internationally traded, with flows of high‐value fresh and frozen products predominantly from poorer to richer nations (Troell et al., 2014), most production in the leading aquaculture countries, almost all low‐ and middle‐income countries, is consumed domestically (Belton, Bush, & Little, 2018). Production of both hatchery‐derived and self‐recruiting species, including small indigenous species, by smallholders in ponds and rice fields in rural Bangladesh is a key part of the harvest directed to both local markets and household subsistence, improving local food security (Haque, Little, Barman, Wahab, & Telfer, 2014; Karim et al., 2011).

In Bangladesh, the dynamics of aquaculture ecosystems in coastal zones are complex, ranging from saline to freshwater aquatic environments, with seasonal and annual fluctuations in freshwater availability (de Roos et al., 2019). Dependency on salinisation and changes in market access and the acceleration of the introduction of new technologies have made the aquatic farming systems highly dynamic, characterised by the integration of varying combinations of freshwater prawns, rice, fish, vegetables and brackish water shrimp (Faruque et al., 2017). Whereas salinisation have stimulated these diverse integrated systems, thereby reducing the risk and vulnerability of farming households, the agrobiodiversity is actually decreased at higher salinity levels (Faruque et al., 2017).

How exactly these agricultural ecosystems link to the nutritional status of its (local) population is unknown. What we do know is that aquaculture systems are designed to maximise productivity, with currently little consideration for the nutritional quality of fish produced (de Roos et al., 2019). Indeed, policies that address the importance of increasing production and processing in the fisheries subsectors in an environment‐friendly and sustainable manner often fail to address its impact on human diet and health outcomes (Bangladesh Country Investment Plan, 2011; Bangladesh Delta Plan 2100, 2017; National Food Policy, 2006). We are aware of silent physical threats, such as soil and river salinity and arsenic contamination, having direct and indirect effects on agricultural production and households' access to food in Bangladesh (Ayers et al., 2017). Furthermore, from a production perspective, farmed species that are most affordable or desired, such as carp, are often lower in levels of LC n‐3 PUFA (Thilsted et al., 2016). The lower overall nutritional quality of farmed fish is illustrated by the fact that in Bangladesh, intake of iron and calcium from fish has significantly decreased in the past two decades, despite a 30% increase in fish consumption (Bogard et al., 2017a). Indeed, nonfarmed fish currently contribute to greater micronutrient intakes than farmed fish in rural Bangladesh (Bogard et al., 2017b). These findings emphasise the urgent need for aquaculture systems to start considering their role in meeting nutritional demands for dietary LC n‐3 PUFA and micronutrients. Indeed, as aquaculture becomes an increasingly important food source, its policies must start considering strategies that ensure the supply of high‐quality and nutrient‐rich fish products in the future. On the other hand, fish consumption has been largely absent from strategies for reduction of micronutrient deficiency (Allison, Delaporte, & Hellebrandt de Silva, 2013). We currently lack food system studies that establish the relationship between fish production strategies and the role of fish consumption in micronutrient intake and deficiency. Such studies would optimise the complementary role that aquaculture and capture fisheries can play in improving nutrition and health, and in particular micronutrient deficiency.

## 4 CONCLUSION

Aquaculture food systems that consider and integrate factors, such as food production, processing and marketing systems, purchasing and eating behaviours, nutritional requirements, access to foods, cultural norms and household composition and behaviours, will enhance our understanding of the role of fish production and distribution on nutritional status and health outcomes of individuals and populations. Although policies that address the importance of increasing production and processing in the fisheries subsectors in an environment‐friendly and sustainable manner mostly fail to address its impact on human diet and health outcomes, the current National Strategy on the Prevention and Control of Micronutrient Deficiencies Bangladesh does acknowledge the impact of nutrition‐sensitive agriculture and food systems on food security. A wider food systems approach that considers the impact of national fish production policies on nutritional status and human health, and the implications of food and health policies for local and national food production systems, will be necessary to develop sustainable strategies that aim to tackle malnutrition and food inequalities. Such an approach will be of special importance for polices that tackle nutrition and health outcomes of vulnerable groups, such as adolescent girls, for whom long‐term improvement in nutritional status will affect not only their own current and future health but also that of the next generation of children.

## CONFLICTS OF INTEREST

The authors declare that they have no conflicts of interest

## CONTRIBUTIONS

BdR, NR, GA, TA, A‐AM, AAS, FM, EG and DCL wrote the review. All authors have read and approved the final manuscript.
